# Expression of eEF1A2 is associated with clear cell histology in ovarian carcinomas: overexpression of the gene is not dependent on modifications at the *EEF1A2* locus

**DOI:** 10.1038/sj.bjc.6603748

**Published:** 2007-04-17

**Authors:** V A L Tomlinson, H J Newbery, J H Bergmann, J Boyd, D Scott, N R Wray, G C Sellar, H Gabra, A Graham, A R W Williams, C M Abbott

**Affiliations:** 1 Medical Genetics, School of Molecular and Clinical Medicine, University of Edinburgh, Molecular Medicine Centre, Western General Hospital, Edinburgh EH4 2XU, UK; 2 Cancer Research UK, Edinburgh Oncology Unit, University of Edinburgh Cancer Research Centre, Edinburgh EH4 2XR, UK; 3 Division of Pathology, University of Edinburgh, Royal Infirmary, Little France, Edinburgh EH4 2XR, UK

**Keywords:** ovarian tumour, clear cell carcinoma, eEF1A2, translation elongation

## Abstract

The tissue-specific translation elongation factor eEF1A2 is a potential oncogene that is overexpressed in human ovarian cancer. eEF1A2 is highly similar (98%) to the near-ubiquitously expressed eEF1A1 (formerly known as EF1-*α*) making analysis with commercial antibodies difficult. We wanted to establish the expression pattern of eEF1A2 in ovarian cancer of defined histological subtypes at both the RNA and protein level, and to establish the mechanism for the overexpression of eEF1A2 in tumours. We show that while overexpression of eEF1A2 is seen at both the RNA and protein level in up to 75% of clear cell carcinomas, it occurs at a lower frequency in other histological subtypes. The copy number at the *EEF1A2* locus does not correlate with expression level of the gene, no functional mutations were found, and the gene is unmethylated in both normal and tumour DNA, showing that overexpression is not dependent on genetic or epigenetic modifications at the *EEF1A2* locus. We suggest that the cause of overexpression of eEF1A2 may be the inappropriate expression of a *trans*-acting factor. The oncogenicity of eEF1A2 may be related either to its role in protein synthesis or to potential non-canonical functions.

Ovarian cancer accounts for 4% of cases of female cancers; it has the highest fatality-to-case ratio of all gynaecological cancers, largely because the vast majority of cases are late-stage at presentation. Tumours arising from the surface epithelium represent the most common form of ovarian cancer. Although a number of molecular mechanisms underlying ovarian tumourigenesis have been identified, a single model of progression has not been described, possibly because of the heterogeneous nature of ovarian carcinoma (Shih Ie and Kurman, 2004). Primary ovarian adenocarcinomas are divided into four common distinct morphological subtypes: serous, mucinous, endometrioid, and clear cell. Although clear-cell tumours are frequently confined to the ovaries at presentation, they are associated with a poor prognosis and are thus treated as high-grade neoplasms (Schwartz *et al*, 2002). The incidence of clear-cell tumours among epithelial ovarian cancers is around 10%. Clear-cell tumours tend to be resistant to platinum-based chemotherapy, giving rise to a poor prognosis (Itamochi *et al*, 2002).

Translation factor eEF1A2 is a tissue-specific variant of eEF1A1 (previously called EF-1*α*). While eEF1A1 is almost ubiquitously expressed, expression of eEF1A2 is normally confined to muscles and neurons (Lee *et al*, 1992; Knudsen *et al*, 1993). The gene encoding eEF1A1 is on 6q13 and eEF1A2 on 20q13.3 (Lund *et al*, 1996). The encoded proteins are 92% identical and 98% similar. Whereas lack of eEF1A2 gives rise to the wasted mouse phenotype (Chambers *et al*, 1998), which involves motor neuron degeneration (Newbery *et al*, 2005), inappropriate overexpression has now been linked to cancer. Anand *et al* (2002) showed that eEF1A2, while not normally expressed in ovary, is expressed in 30% of ovarian tumours . The mechanism for the overexpression appeared to be gene amplification in most, but not all, cases. Anand *et al* (2002) were also able to demonstrate that ectopic expression of eEF1A2 in NIH3T3 cells gives rise to colony formation in soft agar and increases growth rate. Furthermore, overexpression in rat fibroblasts enhances focus formation, and eEF1A2-expressing ES-2 ovarian cells and NIH3T3 cells injected into nude mice give rise to tumour formation. There was however no information on which types of ovarian tumours show overexpression of eEF1A2 and expression was assessed at the RNA level only. eEF1A2 has been identified in an expression microarray study as one of a number of genes that are highly expressed (ca. fourfold) in clear cell ovarian tumours than other histological subtypes of ovarian cancer (Schwartz *et al*, 2002). We recently showed that *EEF1A2* is also a potential oncogene in breast tumours (Tomlinson *et al*, 2005).

We have now set out to examine eEF1A2 expression in a panel of ovarian tumours of defined histological subtypes. We have generated and used a panel of antibodies that allow us to distinguish between the highly related variants eEF1A1 (which is expressed in normal breast and ovary) and eEF1A2 (which is thought only to be expressed only in muscle and neurons under normal circumstances) (Newbery *et al*, in preparation). We show in a panel of ovarian tumours that while a high proportion of clear cell carcinomas overexpress eEF1A2, a far smaller proportion of serous, endometrioid and mucinous tumours have high levels of eEF1A2 expression. Furthermore, we show that (1) DNA copy number at the *EEF1A2* locus is unrelated to expression level, (2) there are no activating mutations in the eEF1A2 coding sequence in tumours where there is overexpression in the absence of gene amplification and (3) the methylation status of the *EEF1A2* gene is unrelated to expression level.

## MATERIALS AND METHODS

### Patient samples: ovarian tumours

Primary ovarian (HOV) tumour material and non-malignant tissues were obtained from patients having undergone gynaecological surgery in the Lothian University Hospitals NHS Trust. Institutional ethical approval was granted for this work by the Lothian University NHS Trust Medicine/Clinical Oncology Research Ethics Subcommittee. Tissue samples were excised and stored in liquid nitrogen. Non-malignant tissue samples were derived from patients who underwent bilateral oophorectomies for suspected malignancy, but were found to have benign histologies; samples were collected from apparently normal contralateral ovaries. Tumours were reviewed by subspecialist gynaecological pathologists, and categorised according to stage and histological type and grade.

### Quantitative real-time reverse transcription–PCR (RT–PCR)

RNA was prepared from ovarian samples and cell lines as described previously (Sellar *et al*, 2003). Total RNA was isolated from tumour and normal tissue using Qiagen RNeasy kits (Qiagen, Crawley, UK). RNA was treated with DNase using DNAfree kit (Ambion, Cambridgeshire, UK) and 1 *μ*g was used for RT–PCR using Retroscript kit (Ambion). TaqMan Assay-on-Demand from Applied Biosystems, Cheshire, UK was used for *EEF1A2* (Assay; Hs 00157325ml) and glyceraldehyde-3-phosphate dehydrogenase (*GAPDH*; control; Hs 99999905ml). In a 10 *μ*l reaction volume per well of a 394-well plate, 0.5 *μ*l of primers, 5 *μ*l of TaqMan PCR Master Mix, no AmpErase UNG 10 × , and 4.5 *μ*l of diluted cDNA were added (Applied Biosystems). Real-time RT–PCR and the quantification of RT–PCR products were performed and the products analysed using an ABI Prism 7900HT Sequence Detection System, and the appropriate software (SDS3.1) according to the manufacturer's instructions (Applied Biosystems).

### Western blots

Protein lysates from cell lines were prepared using previously published protocols (Gilmour *et al*, 2002); the same method was used for primary tumour samples, but in these cases tissue was initially homogenised in extraction buffer before determination of protein content. Western blot analyses using 10 *μ*g protein were carried out using standard protocols. The blots were incubated with primary anti-eEF1A2 rabbit antibody diluted 1 : 200 in blocking solution, as well as primary anti-GAPDH polyclonal mouse antibody (Chemicon International, Hampshire, UK) diluted 1 : 10 000. Blots were then incubated in the appropriate HRP conjugated secondary antibody (Dako Cytomation, Cambridgeshire, UK) at 1 : 500. Detection was performed using enhanced chemiluminescence detection kit (Amersham Biosciences, Buckinghamshire, UK).

### Immunohistochemistry

A computerised search of the archives of the Department of Pathology was used to identify 168 cases of ovarian carcinoma of the common epithelial types. Slides were retrieved and the histological classification reviewed by a gynaecological pathologist. Four representative areas of viable tumour tissue were identified and marked on the slides, and the corresponding areas in the paraffin blocks used as the source of tissue for the tissue microarray (TMA). Tissue cores (0.6 mm diameter) were sampled from each of the four areas, and mounted into separate recipient paraffin blocks by the use of a custom-made instrument (Beecher Instruments, Silver Springs, MD, USA). In the ensuing paraffin array blocks, the tissue cylinders were aligned and marked for identification according to a chart. The recipient TMA blocks were baked at 56°C for 10 min before sectioning, and 3 *μ*m paraffin sections were cut by standard microtomy. A histoarray (CJ1) produced by SuperBioChips (AMS Biotechnology, Oxfordshire, UK) was also used. Formalin fixed, paraffin embedded, sections of human normal tissue, tumour tissue and TMAs were deparaffinised with xylene, rehydrated, treated with picric acid, and microwaved in citric acid at pH 6. Slides were blocked in a 1 : 5 dilution of sheep serum for 30 min at room temperature. Primary anti-eEF1A2 rabbit antibody was used at a concentration of 1 : 10 diluted in phosphate-buffered saline (PBS), for 40 min at room temperature and secondary goat anti-rabbit IgG biotin conjugated antibody (Dako Cytomation, Cambridgeshire, UK) was used at 1 : 200 at room temperature for 30 min. Slides were incubated with StreptABC complex/HRP (Dako Cytomation) at room temperature for 30 min and in diaminobenzidene (Sigma Fast DAB, Sigma, Dorset, UK) for 2 min at room temperature. Alternatively, after the primary antibody step slides were washed in PBS and three drops of ChemMate DAKO EnVision/HRP Rabbit/Mouse secondary antibody (DAKO Cytomation) added to each slide. Slides were incubated for 30 min at room temperature and then washed in PBS. The DAB-containing substrate working solution was prepared by mixing 50 parts ChemMate Substrate Buffer with1 part ChemMate DAB + Chromogen (DAKO Cytomation). One millilitre of this solution was added to each slide and incubated for 5 min. Finally, slides were counterstained in haematoxylin, dehydrated and mounted in pertex.

### Immunohistological scoring methods

The ovarian tumour histoarray (CJ1 SuperBioChips AMS Biotechnology, Oxfordshire, UK) and normal ovarian sections were given a histoscore. For each score, the percentages of the tumour tissue (excluding stroma) which stain strongly (3), moderately (2) and faintly (1) were assessed. The Histoscore was calculated by multiplying the percentage of tumour tissue staining by the score in each category, and adding these values to give a maximum of 300. Expression in the TMAs was assessed using a modification of the method of Tolivia *et al* (2006).

### Statistical methods

Fisher's exact test was used to test for associations between positive protein expression and tumour subtypes of clear cell carcinomas *vs* all other tumour types combined.

### Mutation analysis

For mutation analysis of the *EEF1A2* gene, primer pairs were designed for each exon (see supplementary data). PCR was carried out using *Taq* polymerase from Invitrogen (Paisley, UK), except in the cases of exons 1 and 8, that required the use of DyNAzyme EXT DNA polymerase (Finnzymes, NEB, Hitchin, UK) with cycling conditions consisting of an initial denaturation step at 94°C for 5 min followed by 32 cycles of 30 s at 94°C, 30 s at the annealing temperature and 30 s at 72°C (60 s when using DyNAzyme). PCR products were then sequenced using BigDye v3.1 (Applied Biosystems) according to the manufacturer's conditions.

### Methylation analysis

Methylation analysis was carried out using bisulphite sequencing of a 548 bp region of the *EEF1A2* CpG island. The EZ DNA Methylation Kit (Zymo Research) was used to convert 1 *μ*g of DNA from ovarian tumours HOV 104, 179, 548, and 557 and from normal whole ovary samples 440 and 470 according to the manufacturer's protocol. The resulting DNA was amplified using the following primers: 5′-AGGGATTGGAAATTAGTAGATTT and 5′- AAAAAAAATCCACCTATTAA and Roche Fast Start *Taq* DNA polymerase. Cycling conditions were a 5 min initial denaturation step at 95°C followed by 44 cycles of 95°C for 30 s, 52°C for 30 s, and 72°C for 90 s. The converted PCR products were cloned into the pCR2.1 vector using the TA Cloning Kit (Invitrogen) following the manufacturer's protocol. Clones (2–5 for each original DNA sample) were sequenced as before and the results analysed using BiQ Analyser software (http://biq-analyser.bioinf.mpi-inf.mpg.de/) (Bock *et al*, 2005).

### Quantitative real-time PCR analysis of copy number

For *EEF1A2* DNA copy number analysis two sets of intronic primers were designed (see supplementary data online). Primers designed to amplify microsatellite loci in regions of chromosomes, which are normally stable in ovarian cancers were used for normalisation of total DNA amount. This was based on the method of DNA copy number analysis used by Ginzinger *et al* (2000). For determination of copy number at 20p primers from microsatellite loci on 20p were used and normalised relative to D5S643. Quantitative real-time PCR analysis was carried out using a MyiQ Single Color Real-Time PCR machine (BioRad, Hemel Hempstead, UK) and iQ SYBR Green Supermix (2 ×) (BioRad). Standard curves were conducted on 100 ng DNA extracted from normal blood that was serially diluted five times from 1 : 10 to 1 : 100000. 200 ng of DNA from each tumour was used in a 25 *μ*l reaction. Cycling conditions were an initial 8.5 min denaturing step at 95°C followed by 40 cycles of 95°C for 30 s, and 72°C for 30 s. Fluorescent signal collection was carried out at elongation (72°C). A melt curve was included to confirm primer specificity and minus DNA controls were included to confirm that there was no contamination. Standard curves were used to determine the PCR efficiency for each primer set. Primers were used at 200 nM giving a PCR efficiency of between 90 and 110%. *EEF1A2* copy number was then calculated using the (Pfaffl (2001) method of analysis. The copy number of *EEF1A2* in normal blood DNA was determined using an average value from analysis of five different blood DNA samples. The use of three microsatellite loci for normalisation allowed for the exclusion of outlying values. Copy number at the chromosome 20p microsatellites D20S804 and D20S819 was calculated using the standard curve method of analysis (Applied Biosystems User bulletin #3).

## RESULTS

### Expression analysis of ovarian tumours

**Figure 1:**
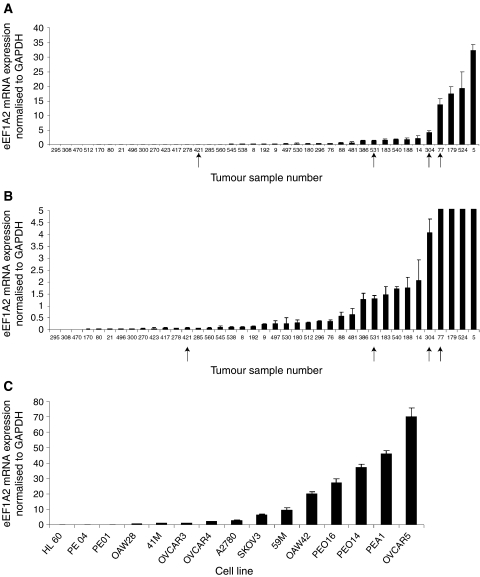
(**A**) Real-time RT–PCR analysis of RNA from ovarian tumours. The amount of eEF1A2 message is shown normalised to GAPDH. Arrows indicate clear cell carcinomas. Samples showing lower levels of expression are shown separately to display small amounts of expression at higher resolution. (**B**) As in (A), but showing the lower part of the scale. (**C**) Real-time RT–PCR analysis of RNA from ovarian cancer cell lines. The amount of eEF1A2 message is shown normalised to GAPDH. The negative control is HL60, a leukaemia cell line.

We assessed RNA and protein samples taken from the same group of ovarian tumours, all of which had been assessed for histological subtype. Initially, we carried out real-time RT–PCR to assess the level of expression of eEF1A2 mRNA in the tumour panel. The results are shown in Figures 1A and B and Table 1; whereas three out of the four clear cell tumours analysed (75%) had detectable overexpression of eEF1A2, only four of the 18 serous tumours (22%), five of the 12 endometrioid tumours (42%) and neither of the mucinous tumours were overexpressing. Similar RT–PCR analysis of a panel of ovarian cancer cell lines shows most cell lines to overexpress eEF1A2 compared with a human leukaemic cell line (HL60), with levels as high as 70-fold higher in the most extreme instance (OVCAR5). The results are shown in Figure 1C.

**Figure 2:**
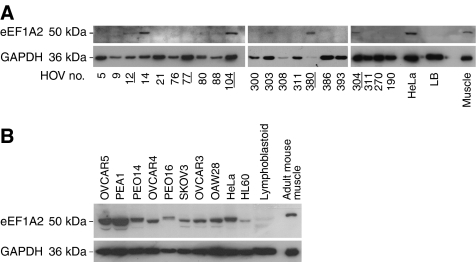
(**A**) Western blot analysis of protein extracts from ovarian tumours. Sample numbers are the same as those shown in Figure 1A. The same blot is shown having been exposed sequentially to eEF1A2 and GAPDH antibodies with a stripping step in between each antibody. The controls are HeLa, which expresses both eEF1A1 and eEF1A2, lymphoblastoid cells (LB) which express only eEF1A1, and adult mouse muscle, which expresses only eEF1A2. Clear-cell tumours are indicated by bold underlined type. (**B**) Western blot analysis of protein extracts from ovarian cancer cell lines. The negative control is a lymphoblastoid cell line and the positive controls are HeLa, which we have previously found to express eEF1A2 at high levels, and mouse muscle.

We went on to use anti-peptide antibodies (Newbery *et al*, in preparation) against eEF1A2. The antibodies were raised against synthetic peptides, which were designed to maximise the differences between eEF1A1 and eEF1A2. Western blots are shown in Figure 2A. eEF1A2 protein is expressed at detectable levels in most clear cell tumours (four out of five tumours analysed; 80%), but only one out of 23 serous papillary tumours and two out of 11 endometrioid tumours. None of the five tumours with a mucinous component had detectable eEF1A2 expression. Six of the seven clear cell tumours analysed in total were overexpressing at either the RNA or protein level or both. Unfortunately, only a few tumours had both RNA and protein extracts available; of these, only one (14) was positive at both RNA and protein levels; s 308, 80, 21, and 300 were negative at both RNA and protein level and s 9, 76, 88, 386, 77, and 5 had detectable expression at the RNA level but not at the protein level. In this set of tumours, the expression of eEF1A2 is highly significantly associated with the clear cell carcinoma subtype when compared with all other tumour sub-types combined (*P*<0.0012, Fisher's exact test). The eEF1A2-positive tumours represent less than 16% of our total tumour panel, in contrast to the 26% figure obtained by Anand *et al* (2002). This may simply represent the different frequency of clear cell neoplasms in the two sets of samples, or reflect the higher numbers of tumours that are positive at the RNA level, but which do not express the protein. This may suggest that the mechanism of overexpression of eEF1A2 in the tumours does not necessarily lead to the production of a stable protein.

When we examined expression of eEF1A2 by Western blotting in ovarian cancer cell lines we again found most cell lines to show high levels of expression. The results are shown in Figure 2B. This is in contrast with the results of Anand *et al* (2002), who found overexpression in 4 out of 13 cell lines, but is in agreement with previous results in our lab where we found most transformed cell lines to express eEF1A2 (Tomlinson *et al*, 2005) and the study by Joseph *et al* (2004) showing eEF1A2 expression in nine out of 10 cancer cell lines examined. The size of the eEF1A2 band appears to shift slightly from one sample to the next suggesting the possibility of post-translational modifications that vary between samples.

**Figure 3:**
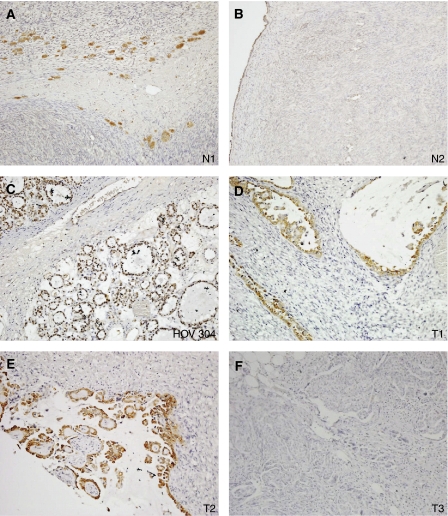
Immunohistochemistry of normal ovary (**A**, **B**) and ovarian cancer (**C**–**F**) sections. Staining in the normal ovary can be seen to be confined to luteinised stromal cells (N1) and low level expression in the ovarian surface epithelium (N2). HOV304 corresponds to one of the clear-cell tumors that was also analysed by RT–PCR and T1 and T2 are clear-cell tumours from the histoarray. T3 is a negative staining tumour. Magnification × 10.

We then carried out immunohistochemistry. We wanted to extend the Western blot analysis, but also to establish whether there is any detectable expression in any cell type in the normal ovary. Figure 3 shows the results obtained with normal tissue. The only cells that were expressing eEF1A2 were luteinised stromal cells, and the ovarian surface epithelium (OSE); there was no staining in the secondary only control. We then analysed a commercial TMA with 57 analysable tissue cores, and a TMA constructed from local cases of ovarian carcinoma as described above, containing a further 91 analysable cores of a single (i.e. not mixed) histological subtype, 148 in total. It can be seen from Figure 3 and Table 2 that the results are largely consistent with those obtained by Western blotting. For the commercial array, although one serous tumour and one mucinous tumour stained moderately, the only epithelial ovarian tumours to display strong staining with the anti-eEF1A2 antibody are clear cell carcinomas. Moderate or strong expression was seen in 60% of clear cell carcinomas by immunohistochemistry on this array. The association between clear cell carcinomas and moderate or strong expression of eEF1A2, in comparison to the other tumour types, was significant (*P*=0.03, Fisher's exact test). For the in-house array a less strong correlation with histological subtype was seen. Only one tumour fell into the ‘strong’ category, with a histoscore of 229, and this was a clear cell tumour, but there were far more negatively and weakly staining tumours overall; the total percentage of tumours with moderate or strong histoscores was 5% for this TMA in contrast to the 19% for the commercial TMA. Nevertheless, when the scores were combined, all three of the tumours with a histoscore of >200 were clear cell carcinomas.

### DNA analysis of the EEF1A2 locus in ovarian tumours

**Figure 4:**
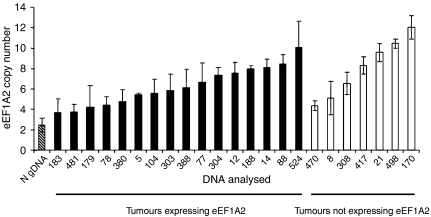
Mean normalised *EEF1A2* copy number in normal gDNA (horizontal stripes), ovarian cancers expressing eEF1A2 at the RNA and/or protein level (black bars), and ovarian cancers that do not express eEF1A2 (white bars). Error bars show 2s.d of the mean.

Anand *et al* (2002) have previously shown that the *EEF1A2* locus is amplified in two out of three tumours they found to be overexpressing eEF1A2, suggesting that amplification of the gene may not be the only mechanism mediating overexpression. We did not have access to cell lines from the tumours we had studied at the RNA and Western level, but did have DNA, so we used a real-time PCR method to estimate the copy number of the *EEF1A2* locus. This was based on the method of Ginzinger *et al* (2000), but control primers were selected from regions that are normally genomically stable in ovarian cancer; three sets were used in case of between-tumour variation. The copy number of *EEF1A2* was estimated using primers within an intron to avoid amplification of *EEF1A1*. We also estimated copy number at two loci on 20p, to distinguish between amplification of 20q and polyploidy. The results are shown in Figure 4; *EEF1A2* was amplified at a significant level (more than 2s.d. from normal DNA) in 12 of the 15 (80%) ovarian cancers expressing eEF1A2 at the RNA or protein level. Only one of these, tumour HOV12, also had an increase in copy number at 20p. However, all eight of those cancers not expressing eEF1A2 also had copy numbers significantly exceeding that found in normal diploid blood DNA. It is clear that there is no obvious correlation between copy number and gene expression; indeed, a non-expressing tumour, number HOV170, shows the greatest *EEF1A2* copy number. The similar pattern of copy number changes between non-expressing and expressing tumours suggests that gene amplification is not the primary mechanism underlying the overexpression of eEF1A2.

**Figure 5:**
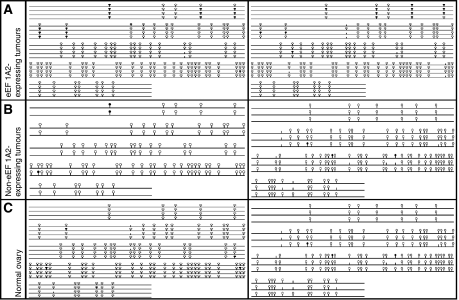
‘Lollipop’ diagrams produced by BiQ Analyser. Open circles denote unmethylated CpG dinucleotides, while closed circles denote methylated CpG dinucleotides. (**A**) Tumours 179 and 104, which do express eEF1A2 and show some methylation in CpG dinucleotides preceding the island, but no methylation within the CpG island. (**B**) Tumours 308 and 470 which do not express eEF1A2. There are very few methylated CpGs either within the island or preceding it. (**C**) DNA from two different normal whole ovaries. There is little methylation present at any CpG dinucleotide within the sequence.

We therefore, went on to establish whether the methylation status of the *EEF1A2* gene differs between ovarian tumours and normal ovarian tissue. We used bisulphite sequencing to compare the methylation profile of 548 bp of the CpG island at the 5′ end of the *EEF1A2* gene in DNA samples from normal ovary, from tumours that do not express eEF1A2 and from overexpressing tumours. It can be seen in Figure 5 that although the CpG dinucleotides immediately 5′ to the CpG island were methylated, the CpG island itself was unmethylated in all cases, suggesting that hypomethylation of the *EEF1A2* gene in tumours is not a mechanism for overexpression.

Finally, we sequenced the coding and untranslated regions of the *EEF1A2* gene in those tumours, which were overexpressing eEF1A2, but which had low copy number (and were thus amenable to analysis by sequencing without the potential complication of sequence differences between gene copies). This group comprised six tumours in total. All eight exons (including non-coding exon 1 and the 3′UTR in exon 8) were sequenced together with flanking intronic regions spanning splice sites. Apart from two established single nucleotide polymorphisms (SNPs), the only sequence alteration identified was a G to A substitution in exon 1 of tumour 183. This sequence alteration was present on both strands and is not a known SNP; unfortunately DNA from normal tissue from the same individual was not available, so it is not possible to establish whether this is a tumour-specific change. However, given both that exon 1 is noncoding, and that this tumour shows expression of eEF1A2 at the RNA but not the protein level, the mutation is unlikely to be of functional significance in terms of cancer. The mechanism by which eEF1A2 is overexpressed in these tumours thus remains elusive, but it is clearly of interest that overexpression is able to occur in the absence of mutation or demethylation, and that there is no correlation between overexpression and amplification.

## DISCUSSION

We have shown that, depending on the detection method used, up to 75% of ovarian clear cell carcinomas show overexpression of eEF1A2 at the protein level. At the RNA level, we find that 33% of ovarian tumours in total overexpress eEF1A2, which is consistent with the 30% figure reported by Anand *et al* (2002). In contrast to the results of Anand *et al* (2002), we found some expression of eEF1A2 in normal ovary, both in the OSE (from which tumours are thought to arise), and in small nests of luteinised stromal cells. We can not detect eEF1A2 expression in the vast majority of ovarian tumours examined, however, which argues against the idea that tumour overexpression is simply a feature of the cells from which the tumours have arisen. Rather, it seems likely that inappropriate expression of eEF1A2 contributes to tumorigenicity, as shown by Anand *et al* (2002).

The finding that eEF1A2 overexpression at the protein level is often associated with clear cell carcinomas is intriguing. Our results are consistent with, and extend, those obtained from microarray analysis by Schwartz *et al* (2002), who identified eEF1A2 as one of a group of genes that are highly expressed in clear cell carcinomas than other ovarian tumours. It is unclear why eEF1A2 overexpression would be found particularly in clear cell tumours. One possibility is that there is a link with p53; we found a weak but not statistically significant association between eEF1A2 expression and p53 wild-type status in breast tumours (Tomlinson *et al*, 2005), and clear cell carcinomas of the ovary normally express p53. Alternatively, Schwartz *et al* (2002) showed a tendency to upregulate stress response genes in ovarian clear-cell tumours, which may be relevant given that eEF1A2 is a known binding partner of peroxiredoxin Prdx1 (Chang and Wang, 2006).

The availability of a specific antibody that recognises eEF1A2, but not eEF1A1, may be of use in a clinical setting once further clinical correlations have been performed.

We have carried out a number of analyses of ovarian tumour DNA to establish the mechanistic basis of the overexpression of eEF1A2. Although amplification of the gene is seen in many tumours there is no correlation between locus amplification and gene expression, suggesting that this cannot be the sole underlying mechanism of overexpression. Amplification was seen just as frequently in tumours that do not express eEF1A2; it would be of interest to examine the expression levels of other genes within this amplicon. We sequenced the *EEF1A2* gene from a number of tumours with low copy number, but failed to find any mutations that could be activating or otherwise lead to overexpression (although it remains a possibility that there are mutations in regulatory regions of the gene in some tumours); similarly, there was no correlation between methylation status and expression. It is clear therefore that overexpression does not depend on genetic or epigenetic changes at the EEF1A2 locus; we suggest that the overexpression may be mediated by the inappropriate expression of a *trans*-acting factor in certain tumours. Microarray assays could be informative in this regard.

It is still unknown why eEF1A2 should have oncogenic properties in tissues in which the closely related eEF1A1 is already expressed at high levels. It has been suggested that a straightforward increase in protein synthesis capacity could be responsible (Thornton *et al*, 2003). It is also possible, however, that the two eEF1A isoforms have subtly (or even substantially) different non-canonical roles. Certainly, it has been demonstrated in myoblasts that while eEF1A1 is pro-apoptotic (Ruest *et al*, 2002), eEF1A2 is anti-apoptotic. Moreover, it has recently been shown that co-expression of eEF1A2 and peroxiredoxin, Prdx1, renders NIH3T3 cells dramatically resistant to apoptotic death induced by exposure to oxidative stress (Chang and Wang, 2006). There are numerous reports of the cytoskeletal modifying properties of eEF1A1 (Condeelis, 1995; Edmonds *et al*, 1996); again, these might differ from eEF1A1 to eEF1A2 and possibly relate to tumour invasion and propensity to metastasize. Further *in vitro* and clinical correlation studies should shed light on this. In the meantime, *eEF1A2* could provide a useful new diagnostic marker for a sub-set of ovarian tumours, and ultimately a possible target for therapy.

## Figures and Tables

**Table 1:** eEF1A2 expression in ovarian tumours

	**RNA**		**Protein-Western**	
**Tumour type**	**Number analysed**	**% positive**	**Number analysed**	**% positive**
Clear cell	4	75	5	60
Serous	18	22	23	4
Endometrioid	12	42	11	18
Mucinous	2	0	5	0

GAPDH=glyceraldehydes-3-phosphate dehydrogenase. Samples are scored as positive at the RNA level if they have a score greater than 0.5 when normalised to GAPDH (see Figure 1A).

**Table 2:** Immunohistochemistry for eEF1A2 in different ovarian tumour types using a commercial TMA and an inhouse TMA

**Tumor type**	**Number analysed**	**Negative**	**%**	**Weak**	**%**	**Moderate**	**%**	**Strong**	**%**
Clear cell	35	19	**54**	10	**29**	3	**9**	3	**9**
Serous	64	35	**54**	26	**40**	3	**5**	0	**0**
Endometrioid	38	29	**76**	9	**24**	0	**0**	0	**0**
Mucinous	11	6	**54**	4	**36**	1	**10**	0	**0**
									
Total	148	89	**60**	49	**33**	7	**5**	3	**2**

TMA=tissue microarray. A histoscore of 0 is recorded as negative, between 1 and 100 as weak, between 101 and 200 as moderate and over 201 as strong.

## References

[bib1] Anand N, Murthy S, Amann G, Wernick M, Porter LA, Cukier IH, Collins C, Gray JW, Diebold J, Demetrick DJ, Lee JM (2002) Protein elongation factor EEF1A2 is a putative oncogene in ovarian cancer. Nat Genet 31: 301–305 12053177 10.1038/ng904

[bib2] Bock C, Reither S, Mikeska T, Paulsen M, Walter J, Lengauer T (2005) BiQ analyzer: visualization and quality control for DNA methylation data from bisulfite sequencing. Bioinformatics 21: 4067–4068 16141249 10.1093/bioinformatics/bti652

[bib3] Chambers DM, Peters J, Abbott CM (1998) The lethal mutation of the mouse wasted (wst) is a deletion that abolishes expression of a tissue-specific isoform of translation elongation factor 1alpha, encoded by the Eef1a2 gene. Proc Natl Acad Sci USA 95: 4463–4468 9539760 10.1073/pnas.95.8.4463 PMC22512

[bib4] Chang R, Wang E (2006) Mouse translation elongation factor eEF1A-2 interacts with Prdx-I to protect cells against apoptotic death induced by oxidative stress. J Cell Biochem 100: 267–278 10.1002/jcb.20969 16888816

[bib5] Condeelis J (1995) Elongation factor 1 alpha, translation and the cytoskeleton. Trends Biochem Sci 20: 169–170 7610475 10.1016/s0968-0004(00)88998-7

[bib6] Edmonds BT, Wyckoff J, Yeung YG, Wang Y, Stanley ER, Jones J, Segall J, Condeelis J (1996) Elongation factor-1 alpha is an overexpressed actin binding protein in metastatic rat mammary adenocarcinoma. J Cell Sci 109(Pt 11): 2705–2714 8937988 10.1242/jcs.109.11.2705

[bib7] Gilmour LM, Macleod KG, McCaig A, Sewell JM, Gullick WJ, Smyth JF, Langdon SP (2002) Neuregulin expression, function, and signaling in human ovarian cancer cells. Clin Cancer Res 8: 3933–3942 12473609

[bib8] Ginzinger DG, Godfrey TE, Nigro J, Moore II DH, Suzuki S, Pallavicini MG, Gray JW, Jensen RH (2000) Measurement of DNA copy number at microsatellite loci using quantitative PCR analysis. Cancer Res 60: 5405–5409 11034080

[bib9] Itamochi H, Kigawa J, Akeshima R, Sato S, Kamazawa S, Takahashi M, Kanamori Y, Suzuki M, Ohwada M, Terakawa N (2002) Mechanisms of cisplatin resistance in clear cell carcinoma of the ovary. Oncology 62: 349–353 12138243 10.1159/000065067

[bib10] Joseph P, O'Kernick CM, Othumpangat S, Lei YX, Yuan BZ, Ong TM (2004) Expression profile of eukaryotic translation factors in human cancer tissues and cell lines. Mol Carcinogen 40: 171–179 10.1002/mc.20033 15224349

[bib11] Knudsen SM, Frydenberg J, Clark BF, Leffers H (1993) Tissue-dependent variation in the expression of elongation factor-1 alpha isoforms: isolation and characterisation of a cDNA encoding a novel variant of human elongation-factor 1 alpha. Eur J Biochem 215: 549–554 8354261 10.1111/j.1432-1033.1993.tb18064.x

[bib12] Lee S, Francoeur AM, Liu S, Wang E (1992) Tissue-specific expression in mammalian brain, heart, and muscle of S1, a member of the elongation factor-1 alpha gene family. J Biol Chem 267: 24064–24068 1385435

[bib13] Lund A, Knudsen SM, Vissing H, Clark B, Tommerup N (1996) Assignment of human elongation factor 1alpha genes: EEF1A maps to chromosome 6q14 and EEF1A2 to 20q13.3. Genomics 36: 359–361 8812466 10.1006/geno.1996.0475

[bib14] Newbery HJ, Gillingwater TH, Dharmasaroja P, Peters J, Wharton SB, Thomson D, Ribchester RR, Abbott CM (2005) Progressive loss of motor neuron function in wasted mice: effects of a spontaneous null mutation in the gene for the eEF1 A2 translation factor. J Neuropathol Exp Neurol 64: 295–303 15835265 10.1093/jnen/64.4.295

[bib15] Pfaffl MW (2001) A new mathematical model for relative quantification in real-time RT-PCR. Nucleic Acids Res 29: e45 11328886 10.1093/nar/29.9.e45 PMC55695

[bib16] Ruest LB, Marcotte R, Wang E (2002) Peptide elongation factor eEF1A-2/S1 expression in cultured differentiated myotubes and its protective effect against caspase-3-mediated apoptosis. J Biol Chem 277: 5418–5425 11724805 10.1074/jbc.M110685200 PMC2803684

[bib17] Schwartz DR, Kardia SL, Shedden KA, Kuick R, Michailidis G, Taylor JM, Misek DE, Wu R, Zhai Y, Darrah DM, Reed H, Ellenson LH, Giordano TJ, Fearon ER, Hanash SM, Cho KR (2002) Gene expression in ovarian cancer reflects both morphology and biological behavior, distinguishing clear cell from other poor-prognosis ovarian carcinomas. Cancer Res 62: 4722–4729 12183431

[bib18] Sellar GC, Watt KP, Rabiasz GJ, Stronach EA, Li L, Miller EP, Massie CE, Miller J, Contreras-Moreira B, Scott D, Brown I, Williams AR, Bates PA, Smyth JF, Gabra H (2003) OPCML at 11q25 is epigenetically inactivated and has tumor-suppressor function in epithelial ovarian cancer. Nat Genet 34: 337–343 12819783 10.1038/ng1183

[bib19] Shih Ie M, Kurman RJ (2004) Ovarian tumorigenesis: a proposed model based on morphological and molecular genetic analysis. Am J Pathol 164: 1511–1518 15111296 10.1016/s0002-9440(10)63708-x PMC1615664

[bib20] Thornton S, Anand N, Purcell D, Lee J (2003) Not just for housekeeping: protein initiation and elongation factors in cell growth and tumorigenesis. J Mol Med 81: 536–548 12898041 10.1007/s00109-003-0461-8

[bib21] Tolivia J, Navarro A, del Valle E, Perez C, Ordonez C, Martinez E (2006) Application of photoshop and scion image analysis to quantification of signals in histochemistry, immunocytochemistry and hybridocytochemistry. Anal Quant Cytol Histol 28: 43–53 16566279

[bib22] Tomlinson VAL, Newbery HJ, Wray NR, Jackson J, Larionov A, Miller WR, Dixon JM, Abbott CM (2005) Translation elongation factor eEF1A2 is a potential oncoprotein that is overexpressed in two-thirds of breast tumours. BMC Cancer 5: 113 16156888 10.1186/1471-2407-5-113 PMC1236916

