# Supplementary material for: Expression of eEF1A2 is associated with clear cell histology in ovarian carcinomas: overexpression of the gene is not dependent on modifications at the EEF1A2 locus
Source: Br J Cancer. 2007 Apr 17;96(10):1613–20. doi: 10.1038/sj.bjc.6603748 (PMC2359942; doi:10.1038/sj.bjc.6603748)
Supplement: supplementary data [file 6603748x1.ppt]

## Slide 1
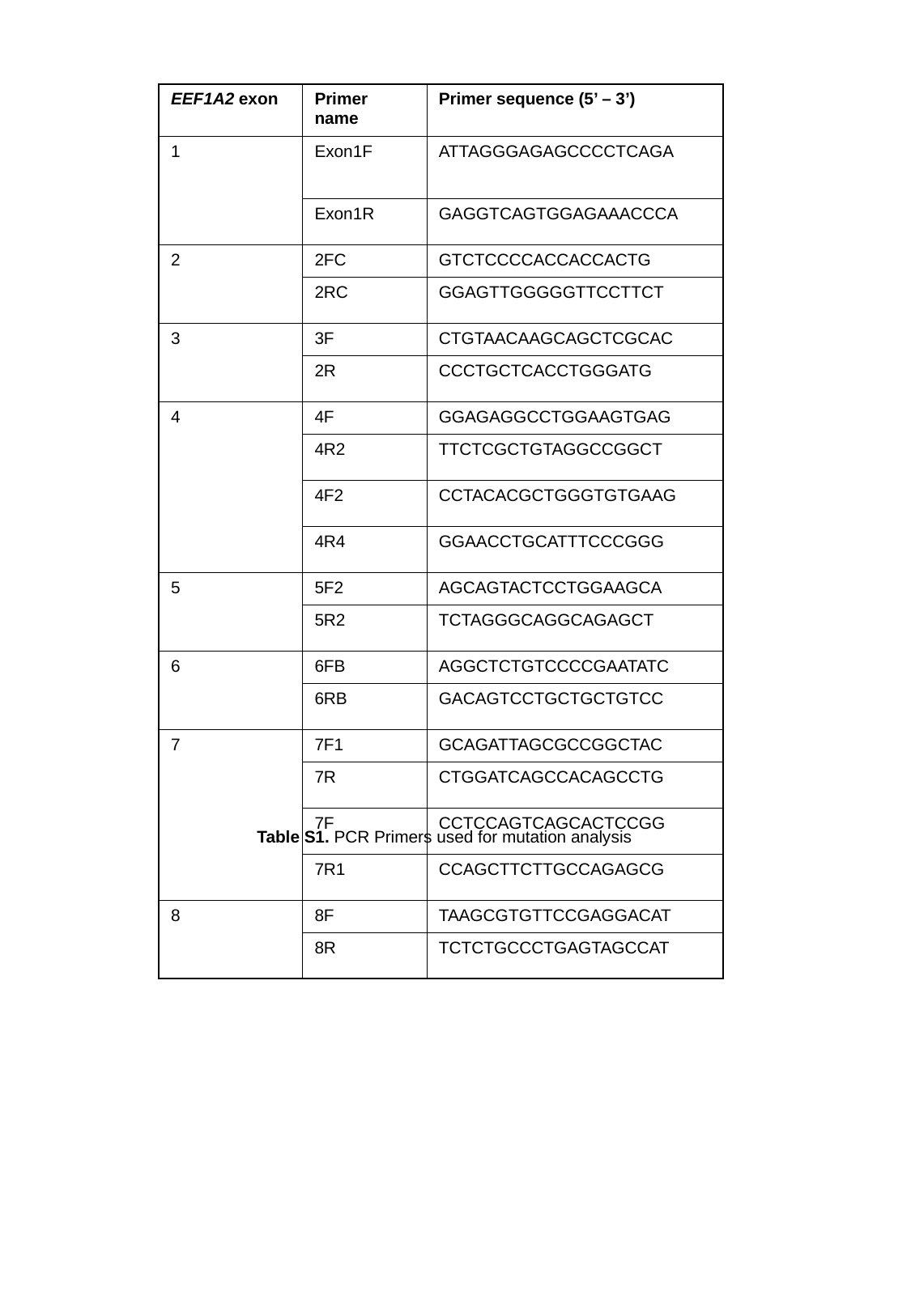

| EEF1A2 exon | Primer name | Primer sequence (5’ – 3’) |
| --- | --- | --- |
| 1 | Exon1F | ATTAGGGAGAGCCCCTCAGA |
| | Exon1R | GAGGTCAGTGGAGAAACCCA |
| 2 | 2FC | GTCTCCCCACCACCACTG |
| | 2RC | GGAGTTGGGGGTTCCTTCT |
| 3 | 3F | CTGTAACAAGCAGCTCGCAC |
| | 2R | CCCTGCTCACCTGGGATG |
| 4 | 4F | GGAGAGGCCTGGAAGTGAG |
| | 4R2 | TTCTCGCTGTAGGCCGGCT |
| | 4F2 | CCTACACGCTGGGTGTGAAG |
| | 4R4 | GGAACCTGCATTTCCCGGG |
| 5 | 5F2 | AGCAGTACTCCTGGAAGCA |
| | 5R2 | TCTAGGGCAGGCAGAGCT |
| 6 | 6FB | AGGCTCTGTCCCCGAATATC |
| | 6RB | GACAGTCCTGCTGCTGTCC |
| 7 | 7F1 | GCAGATTAGCGCCGGCTAC |
| | 7R | CTGGATCAGCCACAGCCTG |
| | 7F | CCTCCAGTCAGCACTCCGG |
| | 7R1 | CCAGCTTCTTGCCAGAGCG |
| 8 | 8F | TAAGCGTGTTCCGAGGACAT |
| | 8R | TCTCTGCCCTGAGTAGCCAT |
Table S1. PCR Primers used for mutation analysis

## Slide 2
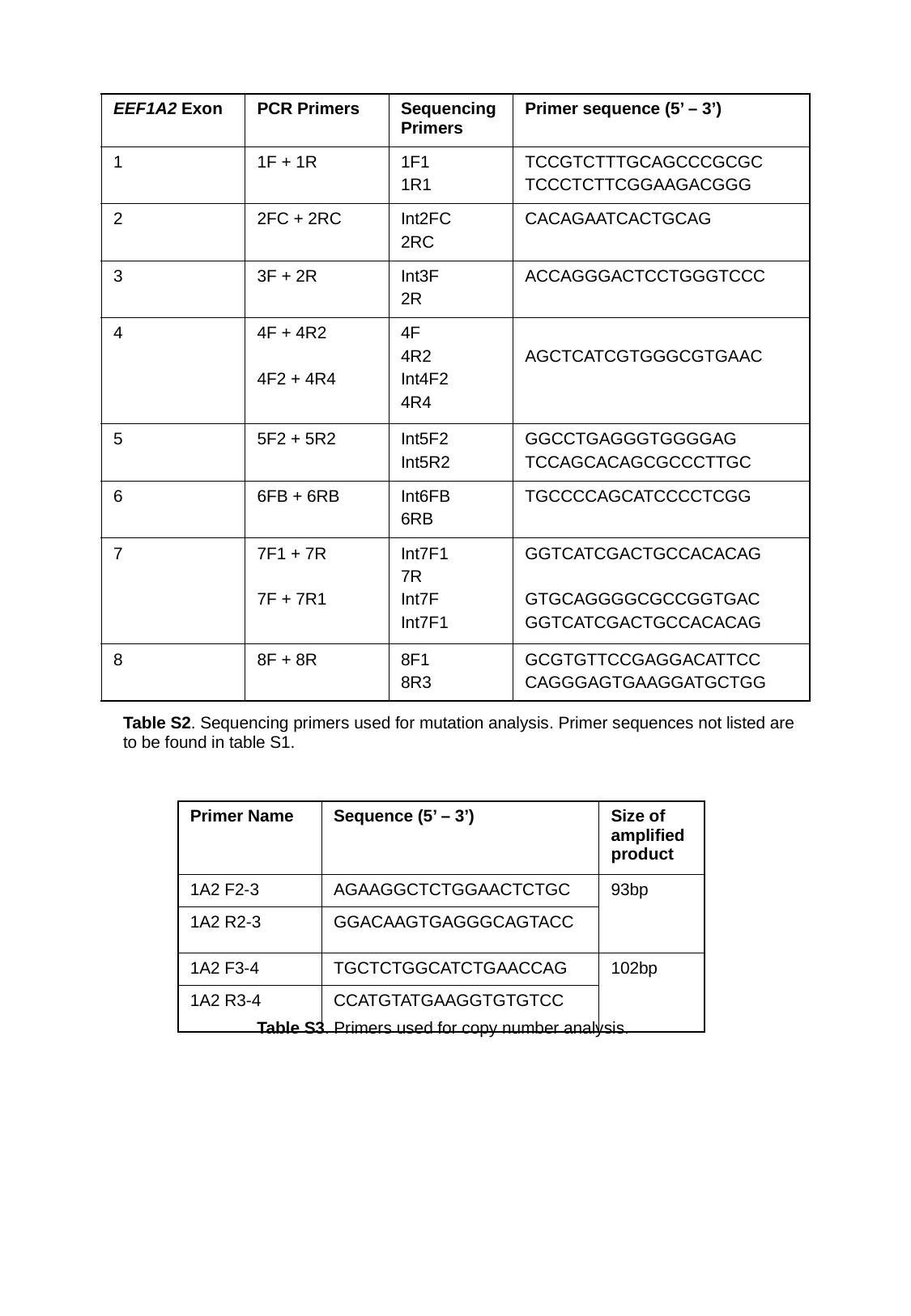

| EEF1A2 Exon | PCR Primers | Sequencing Primers | Primer sequence (5’ – 3’) |
| --- | --- | --- | --- |
| 1 | 1F + 1R | 1F1 1R1 | TCCGTCTTTGCAGCCCGCGC TCCCTCTTCGGAAGACGGG |
| 2 | 2FC + 2RC | Int2FC 2RC | CACAGAATCACTGCAG |
| 3 | 3F + 2R | Int3F 2R | ACCAGGGACTCCTGGGTCCC |
| 4 | 4F + 4R2 4F2 + 4R4 | 4F 4R2 Int4F2 4R4 | AGCTCATCGTGGGCGTGAAC |
| 5 | 5F2 + 5R2 | Int5F2 Int5R2 | GGCCTGAGGGTGGGGAG TCCAGCACAGCGCCCTTGC |
| 6 | 6FB + 6RB | Int6FB 6RB | TGCCCCAGCATCCCCTCGG |
| 7 | 7F1 + 7R 7F + 7R1 | Int7F1 7R Int7F Int7F1 | GGTCATCGACTGCCACACAG GTGCAGGGGCGCCGGTGAC GGTCATCGACTGCCACACAG |
| 8 | 8F + 8R | 8F1 8R3 | GCGTGTTCCGAGGACATTCC CAGGGAGTGAAGGATGCTGG |
Table S2. Sequencing primers used for mutation analysis. Primer sequences not listed are to be found in table S1.
| Primer Name | Sequence (5’ – 3’) | Size of amplified product |
| --- | --- | --- |
| 1A2 F2-3 | AGAAGGCTCTGGAACTCTGC | 93bp |
| 1A2 R2-3 | GGACAAGTGAGGGCAGTACC | |
| 1A2 F3-4 | TGCTCTGGCATCTGAACCAG | 102bp |
| 1A2 R3-4 | CCATGTATGAAGGTGTGTCC | |
Table S3. Primers used for copy number analysis.
